# Characterization of net-zero pozzolanic potential of thermally-derived metakaolin samples for sustainable carbon neutrality construction

**DOI:** 10.1038/s41598-023-46362-y

**Published:** 2023-11-02

**Authors:** K. C. Onyelowe, A. Naghizadeh, F. I. Aneke, D.-P. N. Kontoni, M. E. Onyia, M. Welman-Purchase, A. M. Ebid, E. I. Adah, Liberty U. Stephen

**Affiliations:** 1 Department of Civil Engineering, School of Engineering, University of the Peloponnese, 26334 Patras, Greece; 2 Department of Civil Engineering, Michael Okpara University of Agriculture, Umudike, Nigeria; 3 Department of Civil Engineering, Kampala International University, Kampala, Uganda; 4 Department of Engineering Sciences, University of the Free State, Bloemfontein, South Africa; 5 School of Civil Engineering, University of KwaZulu-Natal, Durban, South Africa; 6 School of Science and Technology, Hellenic Open University, 26335 Patras, Greece; 7 Department of Civil Engineering, Faculty of Engineering, University of Nigeria, Nsukka, Enugu State Nigeria; 8 Department of Geology, University of the Free State, Bloemfontein, South Africa; 9 Department of Structural Engineering, Future University in Egypt, New Cairo, Egypt; 10 Department of Civil and Environmental Engineering, University of Calabar, Calabar, Nigeria; 11 Department of Civil Engineering, University of Agriculture and Environmental Sciences, Umuagwo, Nigeria

**Keywords:** Civil engineering, Structural materials

## Abstract

Metakaolin (MK) is one of the most sustainable cementitious construction materials, which is derived through a direct heating procedure known as calcination. Calcination process takes place substantially lower temperatures than that required for Portland cement, making it a more environmentally sustainable alternative to traditional cement. This procedure causes the removal of hydroxyl water from the naturally occurring kaolin clay (Al_2_Si_2_O_5_(OH)_4_ with MK (Al_2_O_3_·2SiO_2_) as its product. Kaolin naturally exists in large amount within 5°29′N–5°35′N and 7°21′E–7°3′E geographical coordinates surrounding Umuoke, Obowo, Nigeria. Alumina and silica are the predominant compounds in MK, which provide it with the pozzolanic ability, known as the 3-chemical pozzolanic potential (3CPP), with high potential as a cementitious material in concrete production and soil stabilization. Over the years, researchers have suggested the best temperature at which MK is derived to have the highest pozzolanic ability. Prominent among these temperature suggestions were 800 °C (3CPP of 94.45%) and 750 °C (3CPP of 94.76%) for 2 h and 5 h’ calcination periods, respectively. In this research paper, 11 different specimens of Kaolin clay obtained from Umuoke, Nigeria, were subjected to a calcination process at oven temperatures from 350 to 850 °C in an increment of 50 °C for 1 h each to derive 11 samples of MK. The MK samples and Kaolin were further subjected to X-ray fluorescence), scanning electron microscopy (SEM) and X-ray diffraction (XRD) Brunauer–Emmett–Teller (BET) tests to determine the microstructural behaviour and the pozzolanic properties via the 3CPP as to exploit the best MK with the highest cementing potential as a construction material. The results show that the MK heated at 550 °C and 800 °C produced the highest pozzolanic potentials of 96.26% and 96.28%, respectively. The enhancement in pozzolanic potential at optimum calcination temperature is attributed to an increase in the specific surface area upon calcination of kaolinite confirmed by BET results. The SEM and XRD results further supported the above result with the strengthened crystal structure of the MK at these preferred temperatures. Generally, 550 °C is more preferred due to the less heat energy needed for its formulation during 1 h of calcination, which outperforms the previous results, that suggested 750 °C and 800 °C in addition to longer hours of heat exposure.

## Introduction

The concept of carbon neutrality potential in construction materials is gaining prominence in the current construction industry landscape, aligning with the objectives agreed at the COP27 climate change conference^1^. This consensus calls for concerted efforts fromtherole player in the industry to advance the United Nations Sustainable Development Goal 9 (UNSDG9), which is based on the industry, innovation and infrastructure for an attainable net-zero^2^. This has challenged the stakeholders of the built industry to make a swift dive away from the production and utilization of the construction materials with unacceptable greenhouse gas emissions (GHGEs). One of such great efforts have been made with the replacement of the Portland cement with the metakaolin (MK)^3,4^. MK has net-zero properties as it has no carbon footprint. It is derived thermally through hydroxyl water elimination at different temperature exposures with each presenting different aluminosilicate quantities and as such binding potentials.

Due to its eco-friendly potential and highly pozzolanic properties, metakaolin (MK) has been widely investigated. Metakaolin is considered to have twice the reactivity of most other pozzolans and is considered a very viable admixture^3–5^. Metakaolin is produced from calcination of Kaolin clay, one of the most abundant soil mineralsfound in various regions worldwide resulting from the chemical weathering of rocks (feldspar rocks) in hot moist climate^6–13^. Saand et al.^11^ stated that Soorh (a local natural material -Soorh of Thatta District of Sindh, Pakistan) is calcined by an electric furnace at 800 °C for 2 h to produce metakaolin. Metakaolin is acquired by thermal activation of porcelain clay. For a good degree enough thermal activation, the temperature range ought to be between 600 and 750 °C^10^. Metakaolin is a supplementary cementitious material that conforms to ASTM C 618, Class N pozzolan specifications^1^. Sinngu et al.^1^ evaluated South African metakaolin as a cement extender. In their study mortar samples containing varied proportions of Portland cements and metakaolin were subjected several physical, mechanical and durability test along with analytical studies. Based on the results, they suggested that the material meets the criteria to be classified as Class-N pozzolan, as per ASTM C618. Furthermore, it was reported that the best results were obtained by the mixtures containing 15% metakaolin. It is unique in that it is not the by-product of an industrial process nor is it entirely natural; it is derived from a naturally occurring mineral and is manufactured specifically for cementing applications^14,15^. Unlike by-product pozzolans, which can have variable composition, Metakaolin is produced under carefully controlled conditions to refine its colour, remove inert impurities, and tailor particle size^16,17^. Metakaolin is a highly reactive alumino-silicate pozzolan that is rich in silica and alumina. These oxides combine with slake slime Ca(OH)_2_ in the presence of water (moisture) to form compounds that are virtually identical to the compounds in hydrated Portland cement^18^. Metakaolin consists of the mineral kaolinite with a particle size smaller than cement, it is a valuable admixture for concrete forming a high-performance concrete^19,20^. Abiodun et al.^21^ investigated metakaolin obtained as a result of kaolin calcination from some deposits in Nigeria; Ogun (Imeko), Edo (Okpela), Ondo (Ifon) and Ekiti (Isan-Ekiti). They characterized and used it to determine the compressive and flexural strength of metakaolin-based geopolymer concrete (MK-GPC). Most of the abovementioned studies suggest that metakaolin could be used as supplementary cementitious materials to produce materials such as concrete with higher strength, denser microstructure, lower porosity, higher resistance to ions with improved durability properties.

Concrete and soil properties play a very important role in civil engineering construction works particularly in building foundations and road construction works^22^. The stability of foundations and subgrade soils is very essential to the safety and durability of civil engineering structures^23^. One way of improving concrete and soil properties is by stabilization either mechanically or chemically^23^. Concrete production and soil stabilization is mostly at present chemically done using cement and lime. Lateritic soils are one of the important soils and are widespread in tropical areas and subtropical climates^23^. They are the most highly weathered soils in the classification system. The significant features of the lateritic soils are their unique color, poor fertility, and high clay content and lower cation exchange capacity^24^. Pozzolanic materials have the high potential to be used as an economic alternative to lime or cement in stabilization of soils^25,26^. Moreover, these materials can be effectively employed as alternatives to Portland cement as binding agents in concrete, thereby substantially mitigating production costs and the environmental ramifications associated with Portland cement manufacturing^3^. The term pozzolan refers to a siliceous material, which, in finely divided form and in the presence of water, will react chemically with calcium hydroxide to form cementitious compounds^10,11,14^. Pozzolans can be of natural or industrial origin^11,14^. Natural pozzolans include volcanic ash and diatomaceous earth, although pozzolans from industrial by-products are more commonly used today^27^. According to According to ASTM C 618^28^ and Reddy et al.^29^ in order to consider any material as a pozzolan the amount of SiO_2_, Al_2_O_3_, and Fe_2_O_3_ in the chemical compositions of any material should be great than or equal to 70%. ASTM C 618^28^ and Reddy et al.^29^ in order to consider any material as a pozzolan the amount of SiO_2_, Al_2_O_3_, and Fe_2_O_3_ in the chemical compositions of any material should be great than or equal to 70%. Several researches have been conducted with metakaolin in attempt to minimize cost but improve soil and concrete properties for construction purposes.

Zidi et al.^30^ utilized local clay for synthesis and characterization of metakaolin based geopolymers with and without nano-silica. The control geopolymers, for a compressive strength of 30 MPa, were optimized by using Liquid/Solid ratio of 0.55, NaOH concentration of 10 M and curing at 80˚C. The nano silica was added in an extended range of 1%, 2%, 3%, 5%, 7% and 10%. The synthesized nano-silica metakaolin based geopolymers was investigated by using compressive strength, XRD, XRF, FTIR, SEM, MIP, TG, UV/VIS spectroscopy, in addition to density, water absorption and initial setting times. Further research work on metakaolin based geopolymers for soil stabilization was conducted by Samuel in his PhD thesis^31^. Hagrass, et al.^32^ reported that metakaolin as a pozzolanic active material was prepared by firing kaolin ore at different temperatures range from 500 to 1000 °C for different calcination times from 1 to 5 h. The optimum conditions were shown to be at 750 °C for 5 h. At higher temperatures > 900 °C metakaolin undergoes further reactions to form crystalline compounds^32^. Dukuly^34^ investigated the use of metakaolin-based geopolymer as a stabilizing agent for expansive soil. He concluded that this nonconventional approach is an adequate substitute for lime and cement since it increased adequately the unconfined shear strength of soil. Nwaobakata and Ohwerhi^35^ evaluated and predicted the CBR of metakaolin stabilized lateritic soil. They subjected the Kaolin to a calcination temperature of 500 °C to obtain the metakaolin. They conducted preliminary tests such as; Atterberg’s limit test, Specific gravity test and CBR test, using standard experimental procedures on the unmodified soil sample to determine its properties. Scheffe’s simplex theory was used in the development of mix design and optimization model development. The CBR test of modified soil sample was determined and compared with the unmodified counterpart. Their results revealed that the metakaolin improved the properties of the lateritic soil. Reddy et al.^29^ carried out laboratory tests to examine the effectiveness of dissimilar additives Fly Ash, Metakaolin, Fly Ash + Metakaolin combinations, in modifying the expansive soil subgrade properties, thereby improving the strength and reducing the swelling and shrinking phenomenon of expansive soil. They stated that the specific gravity and moisture content of Metakaolin are 2.65 & 0.18% respectively. The bulk density of metakaolin is 710 kg/m^3^ and value of pH is 7. Their result revealed that the optimum values of Maximum dry density was 1793 kg/m^3^, unconfined compressive strength was 0.635 MPa and California Bearing Ratio was 8.96%. They summarize that, fly ash + metakaolin combination (8% + 6%) of admixtures added with expansive soil (black cotton soil) offers potentiality for long term stabilization of expansive soils. Attah et al.^36^ studied lateritic soil treated with metakaolin. Tests carried out included index test, compaction, California bearing ratio and unconfined compressive strength. From their results, it was showed that the use of metakaolin at varying percentages improved the strength properties of the treated soil. Based on their results, they developed a design model for the prediction of CBR values of lateritic soil treated with metakaolin. Pavan^37^ studied experimentally the effect of metakaolin and calcium chloride to improve the properties of expansive soils. Their studies were carried out on the samples of expansive soil, expansive soil + 10% Metakaolin and expansive soil + 10%MK + 1.5% CaCl_2_. It was concluded that addition of Metakaolin (M) and Calcium Chloride (CaCl_2_) reduced the swelling capacity and improved the strength characteristics of expansive soils significantly. This characteristic behaviour shows the improved three chemical pozzolanic potential (3CPP) of the MK upon carboxylation process. The 3CPP is the combined ability of the silica, alumina and ferrite in cementitious construction materials to effectively bind materials through cementation and pozzolanic reaction^15,38,39^. This role takes effect through the formation of cementing gels by calcium-aluminate-hydrate (CAH) and or calcium-silicate-hydrate (CSH)^15,39^.

It is noticed that most of the works on the use of metakaolin have been based on optimizing the compressive strength of concrete. Even though, some researchers have applied metakaolin to soil stabilization of lateritic soil, there is a dearth of literature on characterization and standardization. Beside there is no evidence of characterization and clear standardization of metakaolin as a pozzolanic material in stabilization of lateritic soil in particular and soils in general. What is existing now is more of speculation of the optimum temperature for the most adequate metakaolin. The present study is focused on characterizing the pozzolanic and the microstructural potential of eleven thermally-derived (11 T-derived) metakaolin as a construction material. The approach is based on firing kaolinite clay to temperature range of 350 °C to 850 °C at an increment of 50 °C. Based on these investigations, the study will be able to state at what temperature the desirable and most pozzolanic metakaolin is derived for use as a sustainable construction material in both soil stabilization and greener concrete production.

## Materials and methods

### Materials preparation

**Figure 1:**
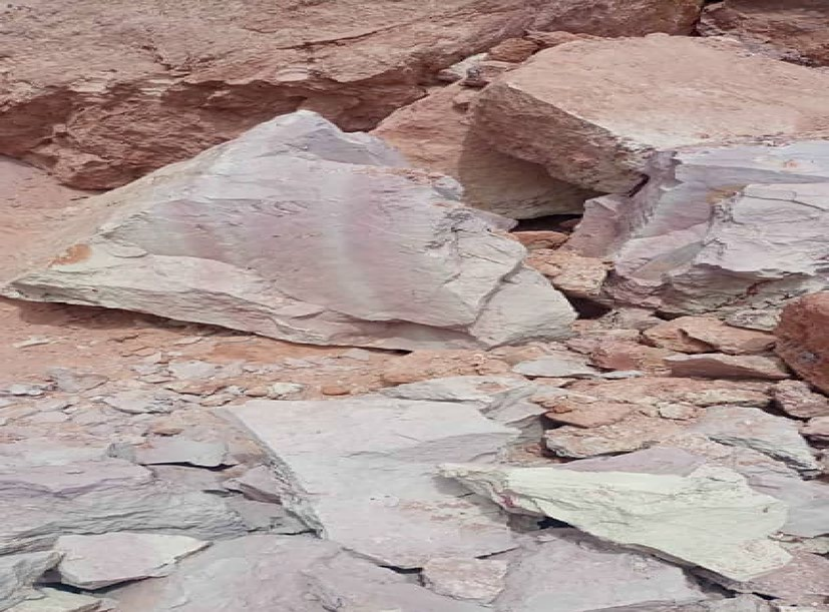
Natural Kaolin collected from a dominant site at Umuoke, Obowo.

**Figure 2:**
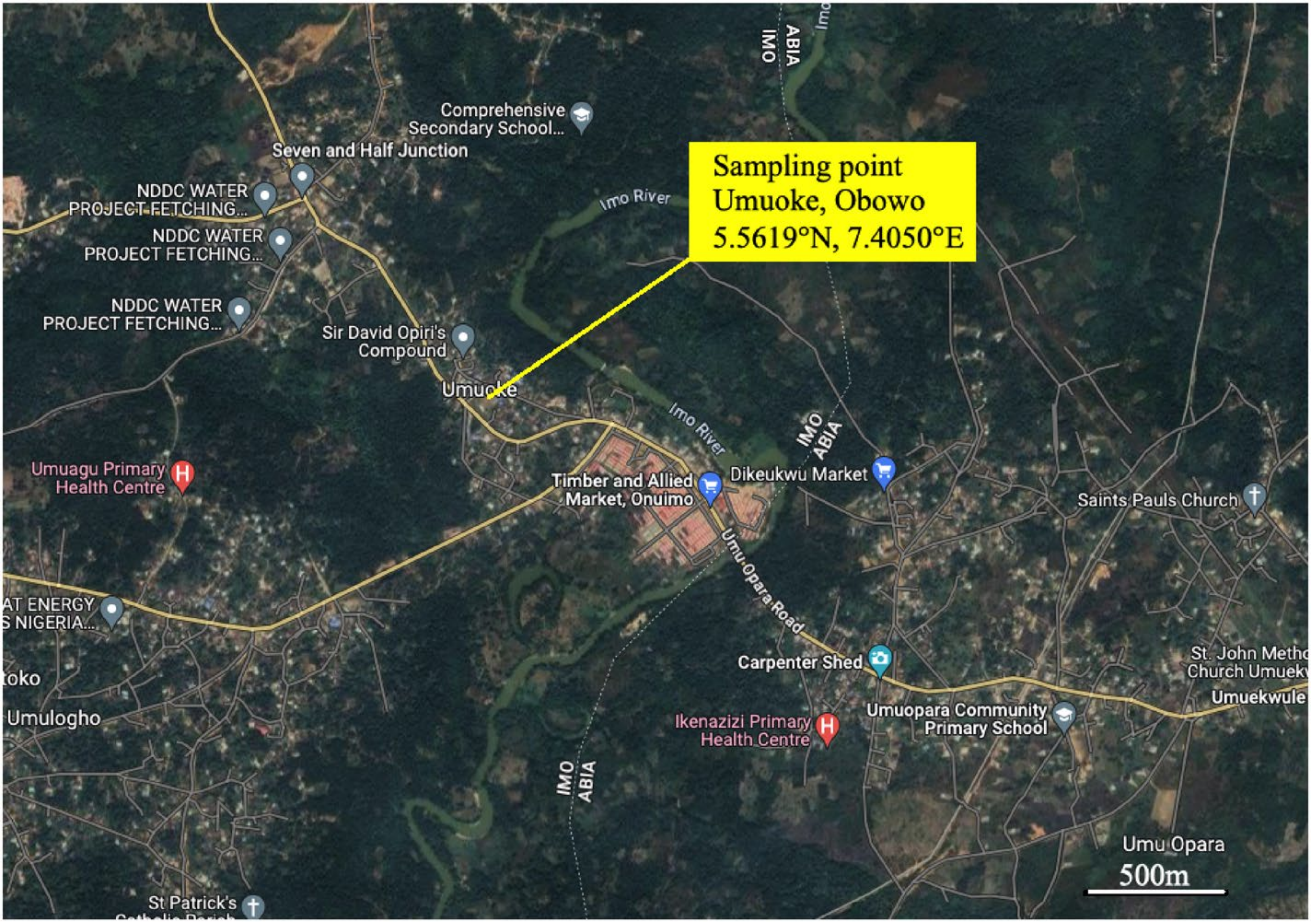
Geographical location of deposited kaolin^40^.

The natural kaolin clay as shown in Fig. 1 was collected from a site located at Umuoke, Obowo in Imo State, Nigeria on coordinates, 5.5619°N, 7.4050°Eas mapped in Fig. 2. The entire community of Umuoke and all surrounding communities extending to Ohiya and Ohuhu in Abia State, Nigeria are all seated on abundant kaolin clay and this naturally occurring mineral is yet to be tapped for use in various engineering and materials productions. The clay material was crushedand 30 g portions of sample were placed in heat-resistant containers. These specimens were subjected to different temperature conditions for 1 h each in a 1000 °C-capacity oven for the calcination procedure.

### Experimental methods

The clay material specimens wereheated in the oven to 350 °C, 400 °C, 450 °C, 500 °C, 550 °C, 600 °C, 650 °C, 700 °C, 750 °C, 800 °C, and 850 °C for 1 h each, in order to remove hydroxyl moisture producingmetakolin (MK) in a dihydroxylation reaction process as shown in Eq. 1.1

The MK specimens were subjected to the X-ray diffraction (XRD) micro-spectral, X-ray fluorescence (XRF) mineralogical and Scanning Electron Microscopy (SEM) microstructural tests in accordance with the appropriate international design and materials standards (BS 1377, 1990). Prior to analysis, all samples were subjected to milling in a carbon steel ring and puck pot. XRD analysis was completed using a “*PANalyticalEmpyrical*” with a Cu-anode X-ray tube, 45 kV and 40 mA. The configuration is Bragg–Brentano geometry focus position along with a Theta-Theta goniometer. High-score was used for interpretation along with the database ICDD-PDF2 2021. Samples were scanned between 3.5° and 70° 2θ. The milling process for XRF is the same as for XRD. Loss on ignition (LOI) was determined by heating the sample to 950 °C, after which a portion of the sample is fluxed and a bead is moulded. A 4 kW WD-XRF Rigaku-Primus IV was used for analysis with a Rh tube. A portion of the original samples were placed on double sided carbon tape on a glass section and carbon coated using a Quorum Q150 sputter coater. A Jeol JSM 6610 Scanning Electron Microscope (SEM) equipped with an EDS (JED-2300 EDS) detector, was used for analysis. The accelerating voltage of the filament is run at 20 kV. Additionally, high resolution images, were also obtained using a JSM-7800F Extreme-resolution Analytical Field Emission SEM. A Brunauer–Emmett–Teller (BET) analysis was also conducted on the Kaolin and a sample of the MK derived at 800 °C to determine the effect of calcination (dihydroxylation) on the specific reactive surface. Finally, the ASTM C618^28^ and BS 8615-1^38^ materials standards were used to characterize and standardize the studied MK specimens for pozzolanic strength based on the micro-spectral, mineralogical and microstructural statuses.

## Results and discussion

### The X-ray fluorescence quantitative analyses of the MK and the 3CPP

The results of the chemical oxide quantitative analyses on both the kaolin and the metakaolin presented in Table 1 show that the dominant compounds present are silica (SiO_2_), alumina (Al_2_O_3_) and ferrite (Fe_2_O_3_). This composition determines the degree of potential cementation a pozzolanic materials shows according to the American and British materials standards for pozzolanas^28,38^. This was judged with the 3-chemical pozzolanic potential (3CPP), which shows a slight increased from 94.32% for the natural kaolin to 94.78% for the 350 °C derived MK. This behavior increased and dropped with different heat exposures but recorded the highest 3CPP values at 550 °C and 800 °C with 96.26% and 96.28% respectively, which shows a difference rate of 0.00021% for an energy need index of 31.25% (a temperature need difference of 250 °C). Considering the energy dissipation needed to achieve an extra 0.02% of cementation potential, the preference should be to make of the MK with 3CPP of 96.26%, which is more sustainable and agrees with Onyelowe and Usungedo^39^ and Ashraf et al.^15^, which reported on the chemical moduli of pozzolanas. But this suggestion is divergent from the views of El. Diadamony et al.^32^, who rather supposed that 750 °C calcination exposure for 5 h for a derived MK be preferred over a 1-h exposure of 550 °C MK achieved in this research paper.

**Table 1:** XRF major elements quantitative analyses of chemical oxides of MK.

XRF major results (wt%)	350 °C	400 °C	450 °C	500 °C	550 °C	600 °C	650 °C	700 °C	750 °C	800 °C	850 °C	Kaolin
SiO_2_	78.66	80.87	82.14	80.49	82.36	81.36	81.42	81.56	82.02	82.31	78.52	85.51
TiO_2_	1.39	1.22	1.22	1.24	1.23	1.23	1.27	1.24	1.18	1.14	1.17	0.81
Al_2_O_3_	14.95	12.77	12.48	13.05	12.62	12.83	13.28	13.05	12.33	12.77	12.20	7.63
Fe_2_O_3_	1.17	1.26	1.18	1.13	1.27	1.12	1.11	1.18	1.23	1.20	1.16	1.17
MgO	0.00	0.00	0.00	0.05	0.00	0.00	0.00	0.00	0.04	0.00	0.15	0.00
MnO	0.01	0.01	0.01	0.01	0.01	0.01	0.01	0.01	0.01	0.01	0.01	0.01
CaO	0.05	0.05	0.05	0.05	0.05	0.05	0.05	0.06	0.09	0.05	2.87	0.05
K_2_O	0.10	0.07	0.07	0.07	0.07	0.08	0.09	0.08	0.09	0.06	0.94	0.03
P_2_O_5_	0.06	0.05	0.05	0.05	0.05	0.05	0.05	0.05	0.05	0.05	0.25	0.03
Total	96.40	96.28	97.18	96.12	97.66	96.73	97.29	97.23	97.03	98.6197.60	98.4397.27	98.0195.25
LOI	5.35	4.42	4.01	4.28	1.61	1.38	0.82	0.90	0.98	1.01	1.16	2.76
3CPP	94.78	94.90	95.80	94.65	**96.26**	95.31	95.81	95.75	95.55	**96.28**	91.88	94.32

3CPP(3-Chemical Pozzolanic Potentials) = SiO_2_ + Al_2_O_3_ + Fe_2_O_3_.

Significant values are in bold.

### The microstructural analyses of the MK

**Figure 3:**
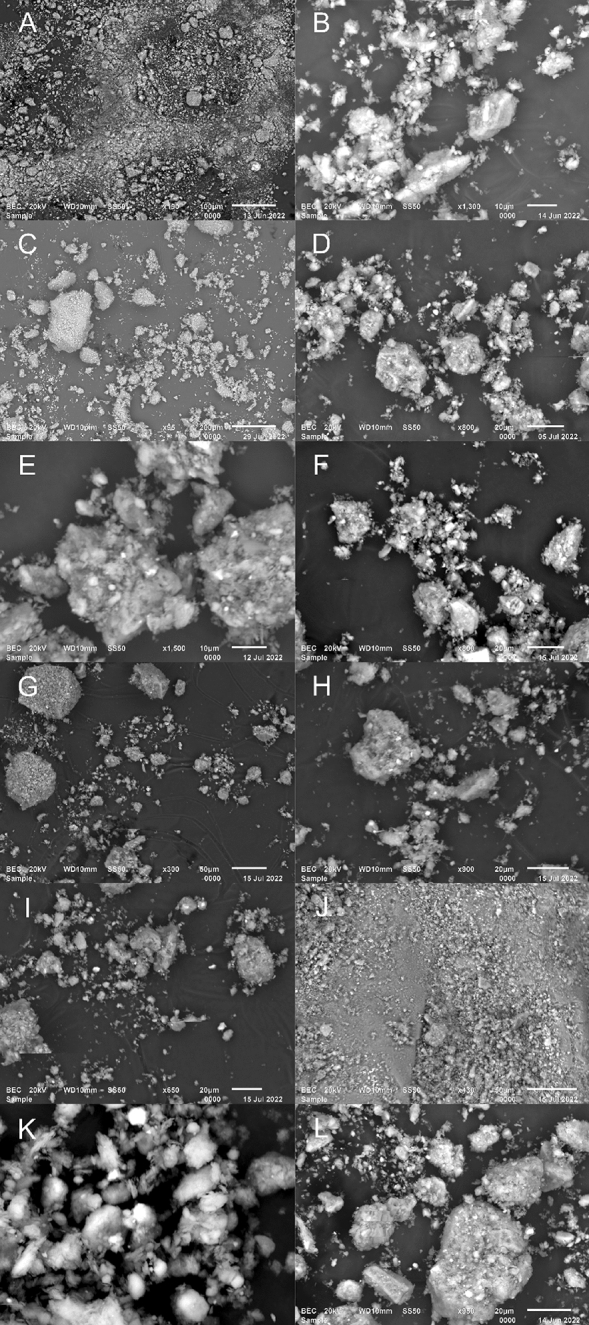
The microstructure of the; (**A**) kaolin and the metakaolin at different dehydroxylation temperatures; (**B**) 350 °C, (**C**) 400 °C, (**D**) 450 °C, (**E**) 500 °C, (**F**) 550 °C, (**G**) 600 °C, (**H**) 650 °C, (**I**) 700 °C, (**J**) 750 °C, (**K**) 800 °C, and (**L**) 850 °C.

**Figure 4:**
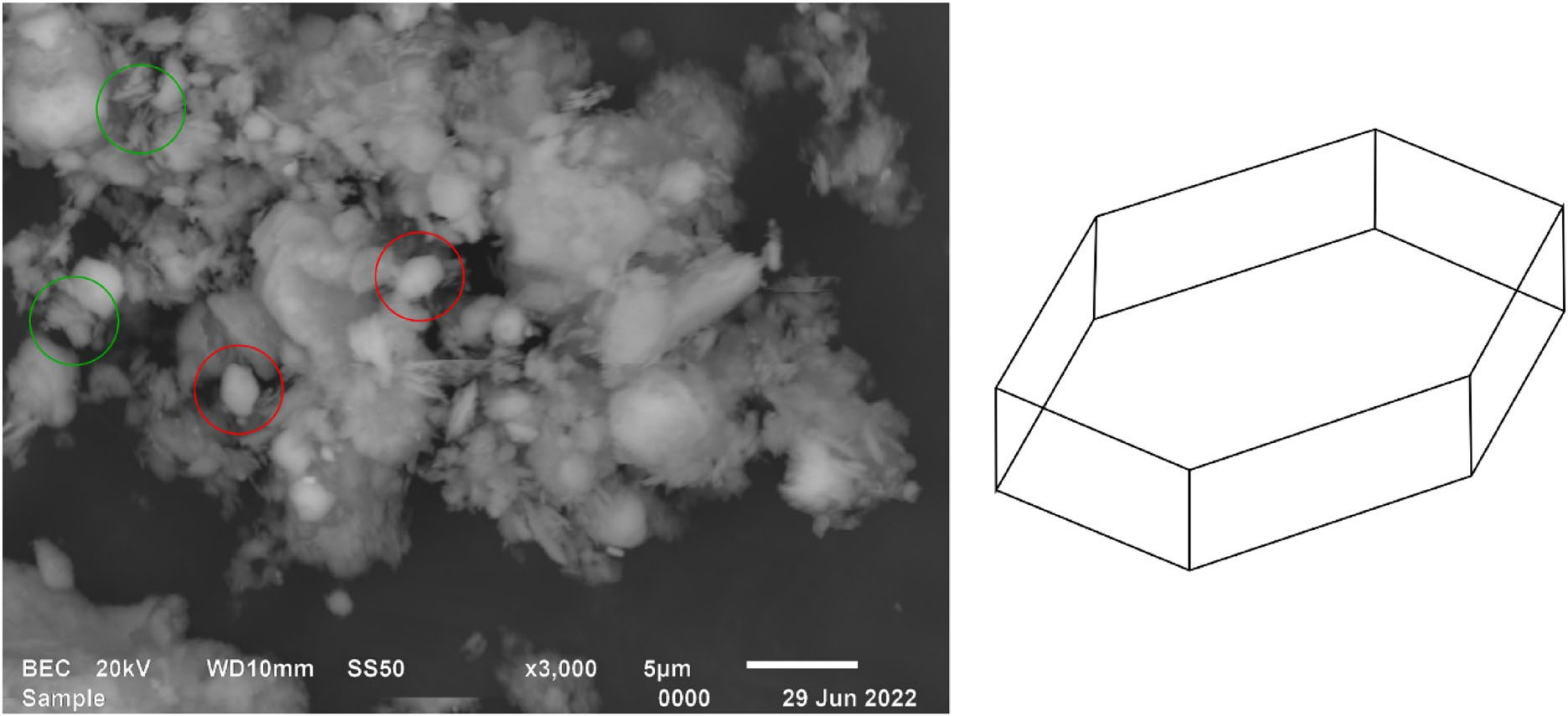
Backscatter electron image (BSEI) of the kaolin sample on the left, which consists mostly of quartz and kaolinite, that has been heated to 400 °C. The basal shape of kaolinite, displayed on the right, can be observed in the BSEI in the red circles. These structures tend to form stacks or alternating tetrahedral and octahedral layers, which can be viewed in the green circles.

Figure 3 shows the progressive changes in surface configuration of the Fig. 3A kaolin and the metakaolin at different dehydroxylation temperatures exposed for 1 h, 3(b) 350 °C, 3(c) 400 °C, 3(d) 450 °C, 3(e) 500 °C, 3(f) 550 °C, 3(g) 600 °C, 3(h) 650 °C, 3(i) 700 °C, 3(j) 750 °C, 3(k) 800 °C, and 3(l) 850 °C. It can be seen that the micropores as observed in the kaolin structure with mineral defects, which influences the thermal stability of the crystal structure of clay minerals were affected by the heat activation process due to the loss of hydroxyl water^41^. The formation of ettringite due to heat introduction became more prominent in the structures of the 550 °C and 800 °C presented in Fig. 3E,K respectively, which agrees with the pozzolanic oxides quantitative analyses of the XRF’s 3CPP. An illustrative and analytical example has been presented in Fig. 4. Kaolinite is a mineral with the chemical formula of Al_2_Si_2_O_5_(OH)_4_, containing ~ 13.9% OH. The internal crystal structure consists of the alternating of two-layers (at a 1:1 ratio), namely a tetraheral layer (consisting of SiO_2_) and a hydrated octahedral layer (consisting of Al_2_O_3_)^42^. These layers are close in proximity, not allowing for the water molecules to connect the octahedral/tetrahedral structures, which is possible in other clay minerals. The stability of the layers in kaolinite with an increase in temperature has been well investigated. This process, referred to as dehydroxylation, begins at a temperature of ~ 420 °C according to Varga^43^, altering the structure of the mineral. According to Civel et al.^41^, all clay minerals contain defects and kaolinite defects affect the thermal stability of the crystal structure. The lower the defect density, the more stable the mineral, resulting in higher temperatures required for dehydroxylation^41^. The release of OH molecules results in the disappearance of the peak at 7.16 Å (d-spacing) in an XRD analysis, resulting in metakaolinite (MK), with profound binding potential at 550 °C and 800 °C.

### The micro-spectral analyses of the MK

**Figure 5:**
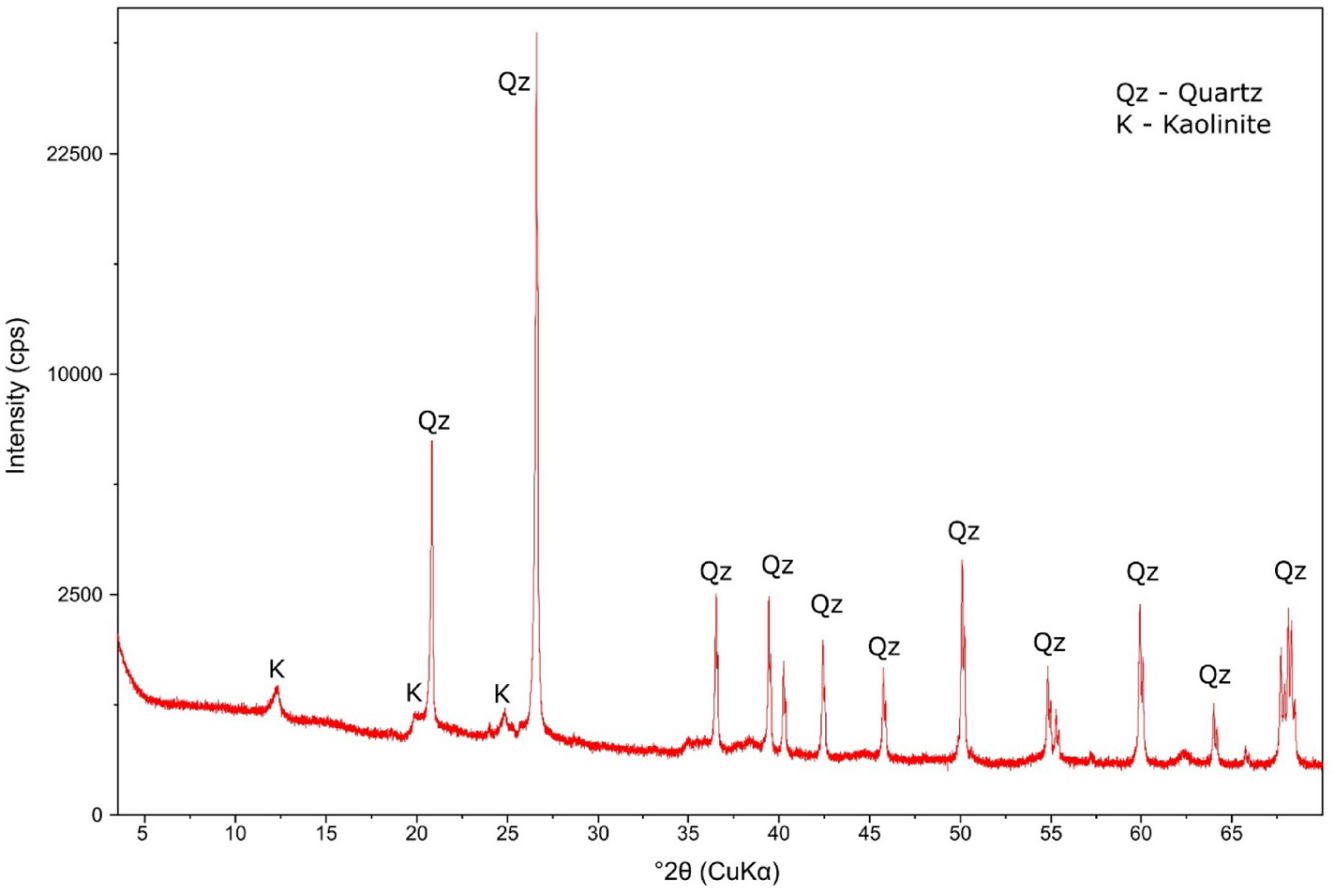
The mineralogical micrograph of the Kaolin.

**Figure 6:**
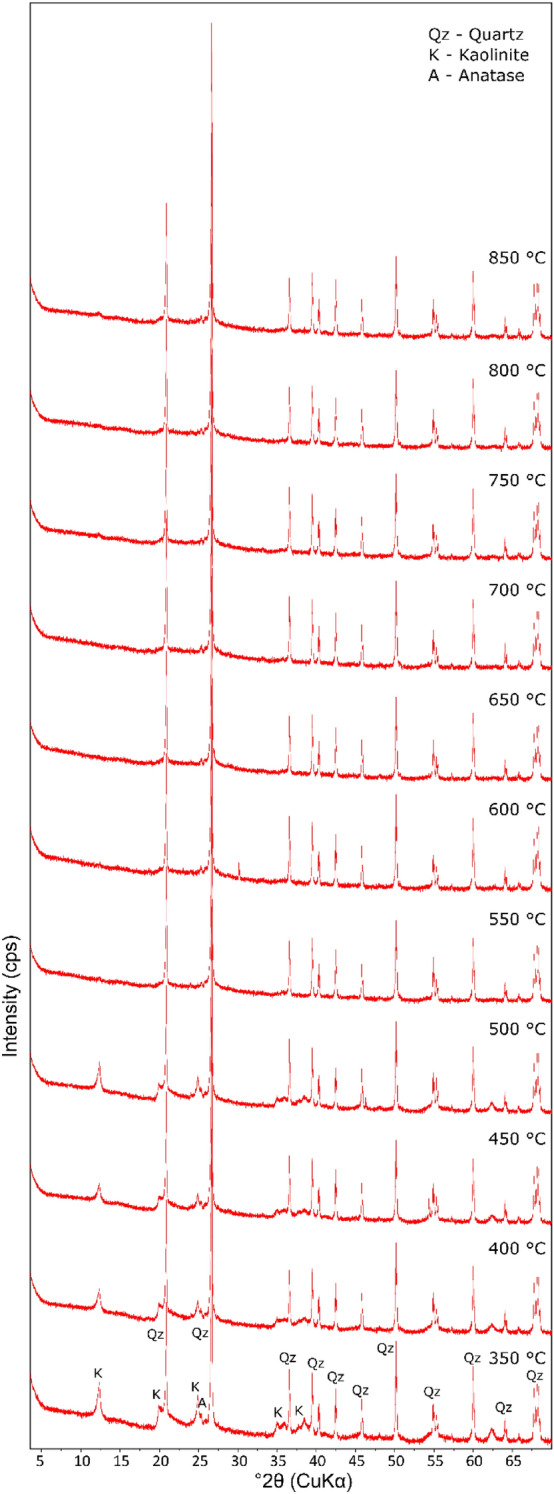
The mineralogical micrograph of the metakaolin at 350–850 °C.

The original kaolin sample’s diffractogram is displayed in Fig. 5 for comparison reasons. The most prominent observation in the diffractograms, obtained through the kaolin dehydroxylation process producingMK, is a metastable mineral anatase (TiO_2_) was introduced by calcination. This phenomenon is present in Figs. 6, which were producedfrom samples at different dehydroxylation temperatures of between 350 and 850 °C and were also compared the control Kaolin sample. This heat-introduced form of TiO_2_ (anatase) mineral is tetragonal in structure, which confirms the origin of the surface structure build in the MK under different calcination temperatures^15^. Also, at the temps of 550 °C and 800 °C, the derived MKs showed more stable quartz, anatase and kaolinite minerals reinforcing the MK pozzolanic ability. The intensity of 2 theta of the quartz minerals increased with higher temperatures. Once again, the MK at 550 °C is preferred due to energy needs. Again, this suggestion is divergent from the views of El. Diadamony et al.^32^, who rather supposed that 750 °C calcination exposed for 5 h for a derived MK be preferred over a 1-h exposure of 550 °C MK achieved in this research paper. As the temperature increased, the peaks corresponding to kaolinite (K) diminished, which may have contributed to the enhanced pozzolanic potential of MK samples, as elaborated earlier in “The X-ray fluorescence quantitative analyses of the MK and the 3CPP” section.

### Brunauer–Emmett–Teller (BET) analysis

**Figure 7:**
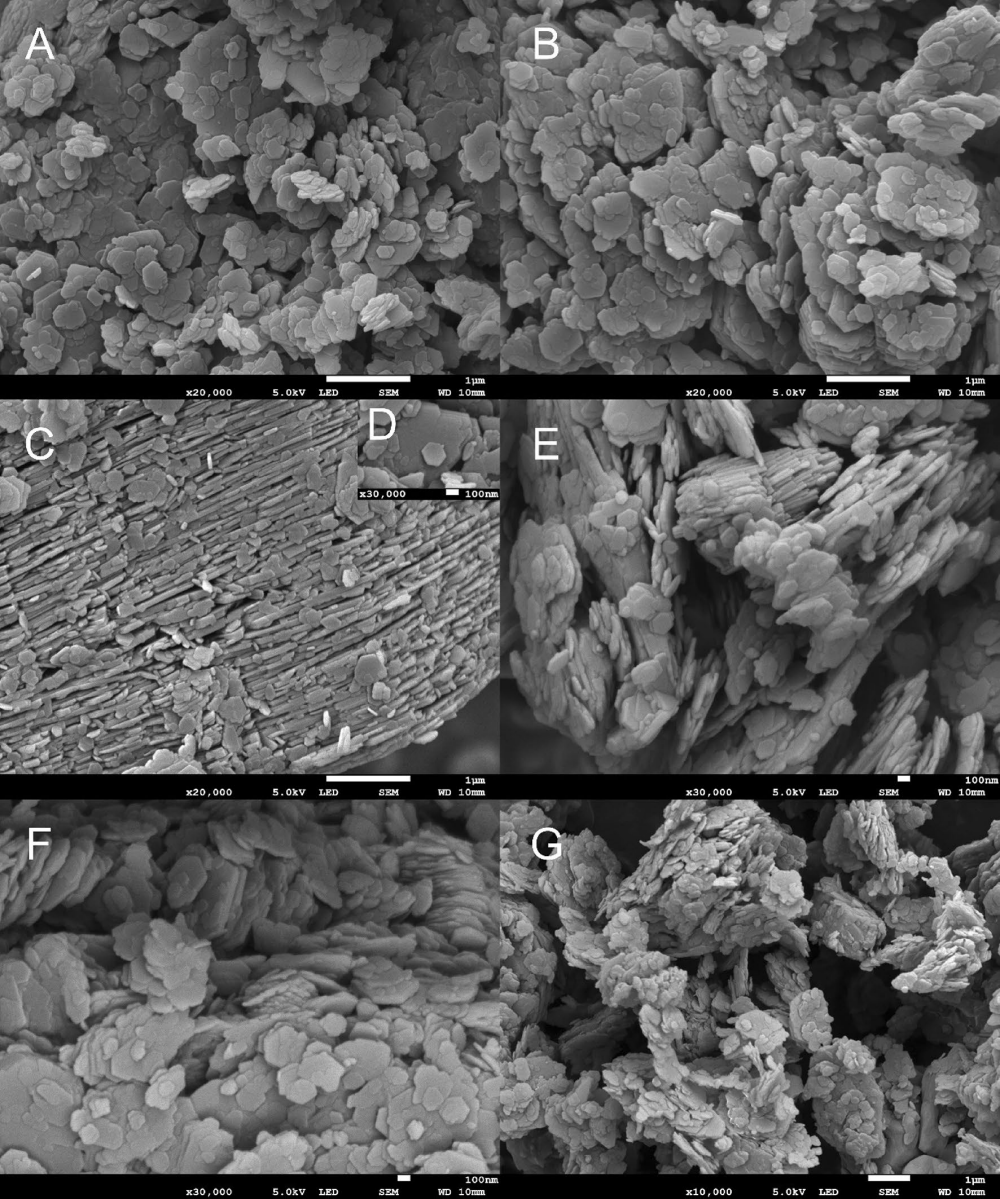
Images from the FESEM analysis. Thicknesses, shape and fringes can be observed. (**A**) Kaolin, (**B**) Result after heating the sample to 350 °C, (**C**) and (**D**) 450 °C, (**E**) 500 °C, (**F**) 700 °C and (**G**) 850 °C.

Moisture content within samples is able to influence the clay behaviour, which is important for engineering purposes^44^. Sample thus goes through thermal treatment. There are two main reactions that occur within kaolinite when heating, namely dehydration and dehydroxylation. Dehydration occurs at ~ 100 °C, which is the removal of physical water^45^. Dehydroxylation occurs between 160 and 500 °C, which removes the OH^-^ molecules from the octahedral layers^45,46^. The second reaction alters kaolinite minerals to amorphous minerals when observed with X-ray methods, yet some structural framework features are preserved^47^. This matter can be seen in the FESEM images presented in Fig. 7, where the structure is preserved as the temperature is increased. The XRD results show that between 500 and 550 °C the kaolinite peaks decrease and eventually disappear. BET analysis was performed on the samples Kaolinite and 800°Cin order to determine the reactive surface area. Prior to the analysis, samples weresubjected to a degassing process, which is this case the parameters chosen for degassing were 105 °C for 16 h. There after it was determined that the reactive surface area of the Kaolinite sample was 6.6973 m^2^ g^−1^ and for the sample 800 °C, 11.4982 m^2^ g^-1^, revealing that the metakaolin samples at optimum temperatures can possess better pozolanic reactivity compared to natural kaolin.

## Conclusions

The three chemical pozzolanic potential (3CPP) of multiple samples of metakaolin (MK) derived under different dehydroxylation temperatures between 350 and 850 °C has been studied with a view to standardize its net-zero cementitious potential based on the mineralogical, microstructural and microspectral behaviours towards carbon neutrality in the construction industry. The behaviour of the natural kaolin and the literature reports were used as baseline for this research work. The following concluding remarks have been drawn;

- The natural kaolin though with a good 3CPP was not preferred as the best supplementary binder due to the mineral defects which affect its long-term performance.
- The MK derived at 550 °C and 800 °C showed the best 3CPP and as such are the best in terms of pozzolanic strength.
- The MK derived at 550 °C with a 3CPP of 96.26% was finally chosen over the one derived at 800 °C due to the less heat energy requirement. It also met the minimum material requirement for pozzolanas according to ASTM and BSI and can potentially replace cement and other carbon footprint-based construction materials especially cementitious ones towards achieving the 2050 carbon neutrality project in the built environment.
- The BET analysis showed a significant increase in the specific reactive surface of the MK with increased dehydroxylation.
- The SEM and XRD results further supported the above result with the strengthened crystal structure of the MK at these preferred temperatures. Generally, 550 °C is more preferred due to the less heat energy needed for its formulation during 1 h of calcination, which outperforms the previous results, that suggested 750 °C and 800 °C in addition to longer hours of heat exposure.
- It is recommended to study the microstructural and pozzolanic behaviour of the natural kaolin under temperatures above 800 °C and up to 1500 °C to establish that no other thermally derived MK produces better characteristics as suggested in this research work.

## Data Availability

The data used in this research work has been reported in this manuscript.
